# Impact of Season, Semen Collection Frequency and Between‐Male Variation on Ostrich Seminal Plasma Composition

**DOI:** 10.1111/rda.70186

**Published:** 2026-02-13

**Authors:** Pfunzo Muvhali, Maud Bonato, Irek Malecki, Kevin Douglas, Pieter Swart, Schalk Cloete

**Affiliations:** ^1^ Directorate Animal Sciences, Western Cape Department of Agriculture Elsenburg South Africa; ^2^ Department of Animal Sciences Stellenbosch University Matieland South Africa; ^3^ Department of Biological Sciences University of Venda Thohoyandou South Africa; ^4^ School of Agriculture and Environment The University of Western Australia Perth Western Australia Australia; ^5^ Department of Biochemistry Stellenbosch University Matieland South Africa

**Keywords:** repeatability, semen characteristics, seminal plasma proteins, sperm quality, *Struthio camelus*

## Abstract

This study evaluated the effect of season, semen collection frequency, and male variation on seminal plasma composition of farmed ostriches. Five South African Black ostrich males (4.47 ± 0.95 years of age) were used in spring 2011 and winter 2012. Semen was collected once daily, three times with an interval of 4 days between collections, followed by three collections at two‐day intervals, and daily for three consecutive days using the dummy female method. Seminal plasma volume was measured with an automatic pipette after semen centrifugation, while seminal plasma protein concentration was determined using the Bradford Coomasie assay. Total seminal plasma proteins were obtained as a product of seminal plasma volume and protein concentration. Semen samples collected in spring had lower total seminal plasma proteins and protein concentration compared to samples collected in winter. Semen collection frequency had no effect on total seminal plasma proteins and protein concentration. Seminal plasma volume was independent of season and semen collection frequency. Lastly, between‐male variation was recorded for all seminal plasma traits, with repeatability estimates for seminal plasma volume, seminal plasma protein concentration and total seminal plasma proteins amounting to 0.51 ± 0.19, 0.33 ± 0.18 and 0.44 ± 0.19, respectively. This study established that higher seminal plasma protein levels are characteristic of winter collections, while frequent semen collection exerts no influence on seminal plasma composition. Between‐male variation and repeatability estimates suggest identification and selection for males with favourable seminal plasma concentration could be achievable in the ostrich.

## 1 Introduction

The ostrich industry is of great financial value to farmers and the economy of the South African Agricultural enterprise (DALRRD 2023). According to Cloete et al. (2012), ostriches have been commercially farmed for more than 150 years in the Southern Cape and the Klein Karoo regions of South Africa, mainly to produce meat, leather, and feathers. The production of ostriches currently relies on traditional mating methods where birds are mated at various male to female ratios ranging from pairs to small colonies (Cloete et al. 1998; Lambrechts et al. 2004). Despite the various combinations of male to female mating ratios, the reproduction of ostriches is still unstable, specifically in relation to the fertility of eggs, hatchability, and chick survival at an early age (Cloete et al. 2001; Brand 2012). The instability of these reproductive measures is a cause of concern towards sustainable and efficient ostrich production and warrants investigations.

Assisted reproduction technology, such as semen evaluation and artificial insemination, may provide a potential opportunity to alleviate reproduction challenges affecting the ostrich industry (Malecki et al. 2008; Muvhali et al. 2022a). Advances towards ostrich semen analysis and artificial insemination have been made following the development of a reliable method for semen collection using the dummy female method (Rybnik et al. 2007). Specifically, the effect of season and frequency of semen collection on semen characteristics in the ostrich has been established with summer, spring, and autumn yielding higher quality ejaculates compared to winter, but poor libido in autumn in the Southern hemisphere, while frequent semen collection does not appear to deplete sperm reserves (Bonato et al. 2011, 2012, 2014; Muvhali et al. 2020). In addition, between‐male variation in semen characteristics has consistently been demonstrated for the ostrich in several studies (see Bonato et al. 2010; Smith et al. 2016; Muvhali et al. 2022b).

Preliminary studies into the biochemical analysis of ostrich seminal plasma, on the other hand, has revealed high total protein concentration and reported variable mineral content between individual males (Ciereszko et al. 2010; Smith et al. 2018a), leading to the development of an ostrich‐specific semen diluent (Smith et al. 2018b). In most avian species, seminal plasma plays crucial roles in sperm function, including cellular, molecular, and metabolic interactions linked to fertility, as well as a protective agent during sperm preservation (Tawang 2017; Santiago‐Moreno and Blesbois 2020). Studies in other bird species, including turkeys, emus, and chickens, have identified multiple factors affecting seminal plasma composition, such as breed and individual‐specific variation between males (Thurston et al. 1982; Tawang 2017; Santiago‐Moreno et al. 2019). In the ostrich, investigations into the biochemical analysis of seminal plasma composition have thus far been limited to two studies with varying semen collection regimes and environmental conditions (i.e., spring in South Africa: see Smith et al. 2018a; and summer in Poland: see Ciereszko et al. 2010). The effect of semen collection days (representing the frequency of collection) was shown not to influence seminal plasma volume, but it influenced certain micro minerals crucial for sperm functionality and reproduction in the South African study by Smith et al. (2018a), while the Polish study did not report on that effect.

Male ostriches produce semen throughout the year with notable seasonal and individual variation (Bonato et al. 2014; Muvhali et al. 2020). It therefore remains necessary to determine whether these factors also influence key semen parameters, such as seminal plasma protein concentration, under similar management and collection conditions (Malecki et al. 2008). Therefore, the aim of the present study was to investigate the effect of season, semen collection frequency, and between‐male variation on ostrich seminal plasma protein concentration.

## 2 Materials and Methods

### 2.1 Study Site and Animals

This study was conducted in the spring of 2011 (September–October) and in the winter of 2012 (June–July) at the Oudtshoorn Research Farm in the Western Cape province of South Africa. The ethical use of animals in this study was approved by the Western Cape Department of Agriculture's Departmental Ethical Committee for Research on Animals (Ref No.: R9/24). A total of five South African Black ostrich males aged between three and 5 years old were used. These birds were maintained in 8 × 6 m individual camps and fed the breeder diet (spring) and maintenance diet (winter) with clean drinking water available ad libitum. The diets were different because September–October coincided with the conventional breeding period on the farm when a breeder diet (10.90 MJ/kg dry matter and 180.9 g/kg protein) was supplied, while June–July coincided with the rest period when a maintenance diet (9.1 MJ/kg dry matter and 133 g/kg protein) was supplied. This led to diet being confounded with season and resulted in the analysis of season as a factor instead of diet. The respective seasons reflected a period of warmer weather with longer photoperiod (spring) and colder weather with shorter photoperiod (winter) in the Southern hemisphere. In addition, these seasons coincide with two periods of opposite sperm quality in the ostrich, with spring reflecting a period of higher sperm quality than winter (Bonato et al. 2014; Muvhali et al. 2020).

The males were trained for semen collection using the dummy female method developed and described in detail by Rybnik et al. (2007). Selection of male ostriches for semen collection training was based on the birds' willingness to interact with a human (Bonato et al. 2013; Muvhali et al. 2023), showing less fear and aggression while cooperating with the human handler to mount the dummy female and ejaculate in the fitted artificial cloaca.

### 2.2 Semen Collection and Assessment

Before the commencement of the experiment in each season, semen was collected twice from all males (6 and 4 days prior to the initial day of the experiment) for synchronicity purposes. Thereafter, three semen collection frequencies (SCF) were evaluated. The SCF included once a day collection three times from all males with an interval of 4 days between collections (SCF1). This semen collection frequency was followed by three collections at two‐day intervals (SCF2), followed by daily collections for a further 3 days (SCF3). Collection of semen was performed in the morning slightly after 08 h 30 AM by the same person.

Semen volume was measured using an automatic pipette. Sperm concentration was determined with a haemocytometer following the dilution of 20 μL semen 1:400 (v/v) with a phosphate buffered saline solution containing 10% formalin. The number of spermatozoa was subsequently calculated by multiplying semen volume and sperm concentration. To separate the spermatozoa from the seminal plasma, semen samples in 1.5 mL Eppendorf microtubes were centrifuged at 654× *g* (3000 rpm, radius 6.5 cm) for 30 min using a Jouan A14 type centrifuge (ST. Herblain, France). An extended centrifuging time was warranted by the high viscosity of ostrich semen, to ensure the complete removal of cell debris and residual sperm. The seminal plasma (supernatant fluid following centrifugation) was measured using an automatic pipette and transferred to new clean labelled 1.5 mL Eppendorf microtubes, while the spermatozoa at the bottom of the centrifuged Eppendorf microtubes were discarded. The seminal plasma was stored at −20°C until required for protein concentration analysis. Upon completion of the semen collection phase, the seminal plasma samples were transported in a frozen state to the Biochemistry Department, Stellenbosch University (approximately 400 km away from the study site) and stored at −80°C until required.

The methodology used by Tawang (2017) was followed to determine seminal plasma protein concentration. The frozen seminal plasma samples were thawed and centrifuged for 30 min at 10,000 rpm at a temperature of 4°C to further remove remaining sperm cells and other debris. An aliquot of 5 μL supernatant was drawn after centrifugation and placed on a microscope slide (20× objective, Olympus BX60 microscope—Olympus Australia Pty. Ltd. Mt. Waverly, VIC, Australia) to verify that the seminal plasma was free of sperm and cell debris. A volume of 10 μL (2 mM) proteolysis inhibitor mixture was added to the supernatant following the final centrifugation to prevent proteolysis. Protein concentration was then measured from all samples as duplicates using the Bradford Coomasie assay (Pierce, Pty. Ltd., Australia). This assay makes use of a colourmetric change where copper forms complexes with proteins, resulting in a colour change from green to purple. A spectrophotometer measuring absorbance at a wavelength of 540 nm allows the determination of the protein concentration (mg/mL) of samples. Total protein concentration in the seminal plasma was calculated as a product of seminal plasma protein concentration (mg/mL) and total seminal plasma volume (mg).

### 2.3 Data Analysis

The data generated from the study was analysed using linear mixed models in ASReml 4.2 (Gilmour et al. 2021), with fixed effects of season and frequency of semen collection. Age of the male was entered as a covariate in the analysis. A square root transformation was applied to normalise response variables that were not normally distributed (semen volume, total seminal plasma protein, seminal plasma volume and total spermatozoa per ejaculate). Repeated sampling of the same bird was accommodated by fitting male identity as a random variable. In another analysis, male identity was fitted as a fixed effect to quantify between‐male variation. In addition, repeatability estimates for the recorded traits were generated. The visualisation of data in this study was done in R‐studio (RStudio Team 2020). Fixed effects were considered significantly different at *p* < 0.05.

## 3 Results

### 3.1 Descriptive Statistics

A total of 80 semen samples were collected, with a mean and standard deviation (mean ± SD) for semen volume, sperm concentration and total number of spermatozoa per ejaculate amounting to 2.02 ± 1.24 mL, 3.38 ± 1.13 × 10^9^ sperm/mL and 6.88 ± 4.80 × 10^9^ spermatozoa/ejaculate, respectively. The mean seminal plasma volume, seminal plasma protein concentration and total seminal plasma protein amounted to 0.92 ± 0.63 mL, 27.75 ± 8.06 mg/mL, and 24.20 ± 15.87 mg, respectively.

### 3.2 Effects of Season and Semen Collection Frequency on Semen Characteristics

Sperm concentration and total number of spermatozoa per ejaculate were higher in semen collected in spring as compared to winter (*p* < 0.05, Table 1). Three consecutive days of semen collection (SFC3) resulted in higher sperm concentration and total number of spermatozoa when compared to the other two semen collection frequencies (*p* < 0.05, Table 1). However, semen volume was not affected by season of semen collection or semen collection frequency (*p* > 0.05, Table 1).

**TABLE 1:** Means (±) standard errors for semen volume, sperm concentration and total number of spermatozoa per ejaculate as influenced by season of semen collection and frequency of semen collection (*n* = 5 South African Black ostrich males).

Season	Semen volume (mL)	Sperm concentration (×10^9^/mL)	Total spermatozoa/ejaculate (×10^9^)
Winter	1.81 ± 0.15	3.14 ± 0.16^b^	5.59 ± 0.56^b^
Spring	2.11 ± 0.16	3.80 ± 0.15^a^	7.86 ± 0.64^a^
*p*‐value	0.113	< 0.001	0.003
*Semen collection frequency*			
SCF1	1.71 ± 0.14	3.09 ± 0.15^b^	5.25 ± 0.51^b^
SCF2	1.97 ± 0.21	3.27 ± 0.20^b^	6.20 ± 0.75^b^
SCF3	2.20 ± 0.22	4.05 ± 0.20^a^	8.83 ± 0.91^a^
*p*‐value	0.363	0.003	0.028

*Note:* ^a,b^Means with different superscript within a row differ significantly (*p* < 0.05). SFC1—once a day semen collection with an interval of 4 days between three collections. SFC2—once a day semen collection with an interval of 2 days between three collections. SFC3—once a day semen collection for three consecutive days.

### 3.3 Effects of Season and Semen Collection Frequency on Seminal Plasma Characteristics

Total seminal plasma proteins and seminal plasma protein concentration were lower in ejaculates collected in spring as compared to ejaculates collected in winter (*p* < 0.05, Table 2). The frequency of semen collection did not affect total seminal plasma proteins and seminal plasma protein concentration (*p* > 0.05, Table 2). In addition, seminal plasma volume was independent of season of semen collection and semen collection frequency (*p* > 0.05, Table 2).

**TABLE 2:** Means (±) standard errors for seminal plasma volume, seminal plasma protein concentration and total seminal plasma proteins as influenced by the season of semen collection (*n* = 5 South African Black ostrich males).

Traits	Season		*p*
	Winter	Spring	
Seminal plasma volume (mL)	0.88 ± 0.08	0.88 ± 0.07	0.912
Seminal plasma protein concentration (mg/mL)	31.79 ± 1.02^a^	22.46 ± 1.00^b^	< 0.001
Total seminal plasma protein (mg)	26.84 ± 2.20^a^	18.64 ± 1.80^b^	0.01

*Note:* ^a,b^Means with different superscript within a row differ significantly (*p* < 0.05).

### 3.4 Between‐Male Variation on Semen and Seminal Plasma Characteristics

**FIGURE 1:**
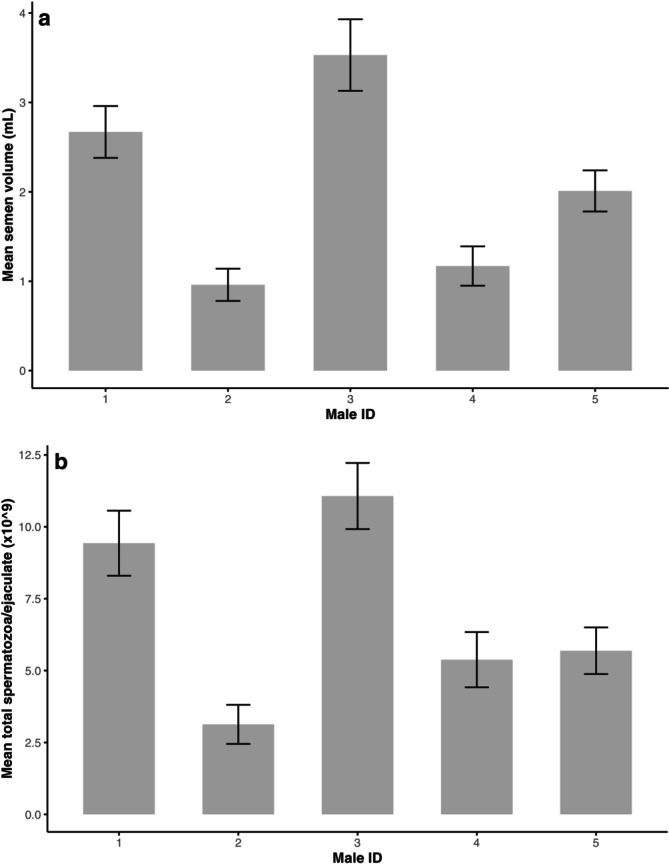
Means (±) standard errors for between‐male variation in (a) semen volume and (b) total spermatozoa/ejaculate for the ostrich (*n* = 5 South African Black ostrich males; *p* < 0.001).

**FIGURE 2:**
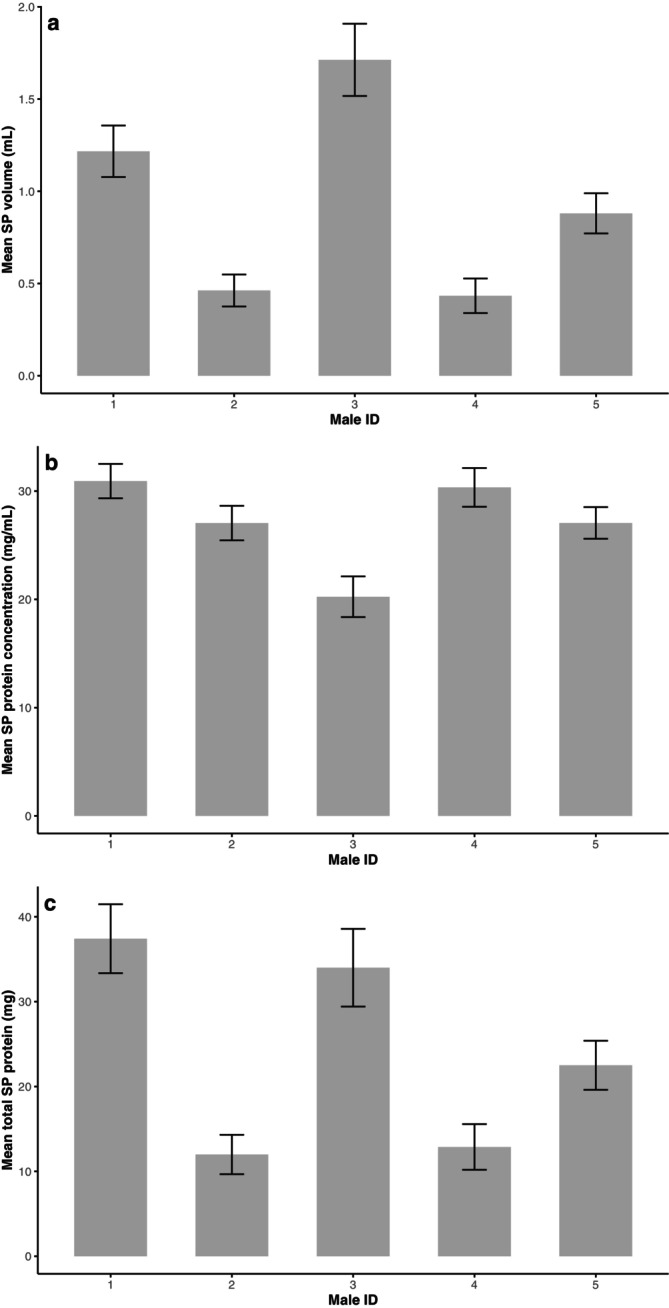
Means (±) standard errors for between‐male variation in (a) seminal plasma (SP) volume, (b) seminal plasma protein concentration, and (c) total seminal plasma protein for the ostrich (*n* = 5 South African Black ostrich males; *p* < 0.001).

There were significant differences between males in semen volume and total spermatozoa per ejaculate (*p* < 0.001, Figure 1), but not sperm concentration (*p* > 0.05). In addition, all seminal plasma characteristics measured in this study differed between males (*p* < 0.001, Figure 2).

Regarding semen characteristics, a low repeatability estimate (0.02 ± 0.06) was derived for sperm concentration, while moderate to high repeatability estimates were derived for the total number of spermatozoa per ejaculate (0.28 ± 0.17) and semen volume (0.47 ± 0.21). For seminal plasma characteristics, repeatability estimates were moderate, ranging from 0.33 ± 0.18 for seminal plasma concentration, to 0.44 ± 0.19 for total seminal plasma protein and 0.51 ± 0.19 for seminal plasma volume.

## 4 Discussion

The current study demonstrated that ostrich sperm concentration and total number of spermatozoa per ejaculate were significantly higher in spring compared to winter. Additionally, these traits were elevated following semen collection over three consecutive days. While the former traits appeared to be influenced by season and frequency of semen collection, semen volume was independent of both factors in the current study. The findings that sperm concentration and total number of spermatozoa were highest in ejaculates collected in spring compared to winter are consistent with previous studies by Bonato et al. (2014) and Muvhali et al. (2020) where ostrich semen was collected throughout the entire year or across all seasons while birds were supplied the same breeder diet. It was impossible in this study to partition the effects of season and diet, but the concurrence of this study with the cited studies controlling for diet does suggest that season may have an overriding effect.

A study by Bonato et al. (2011) utilised various semen collection frequencies and revealed that only twice‐daily semen collection frequency with a 6‐h interval yielded higher semen volume and total number of spermatozoa per ejaculate as compared to other collection frequencies (once every 24 and 48 h). The lack of a semen collection frequency effect on semen volume in this study implies that the frequencies of semen collection used were not intense enough to stimulate higher semen volume as reported previously, while spermatozoa output per ejaculate could be impacted already by single‐daily ejaculation following three consecutive days of semen collection.

Interestingly, seminal plasma protein concentration and total seminal plasma proteins were higher in ejaculates collected in winter and lower in spring, showing an inverse relationship with sperm concentration and total sperm output. Although the mechanism behind this inverse relationship is unclear, this pattern is consistent with findings in ducks, where lower seminal plasma protein concentrations were observed in drakes categorised as high‐quality semen producers based on sperm quality factor indicators (a product of semen volume, sperm concentration, live and morphologically normal sperm) compared to those with lower semen quality (Liu et al. 2008; Tang et al. 2022). In emus (another commercially farmed ratite species), sperm functionality post‐cryopreservation of poor freezer males was improved by the addition of seminal plasma from males considered as good freezers (Tawang 2017). This implies that seminal plasma components, such as proteins and minerals in emu semen could offer protective advantages to sperm, more specifically under thermal stress conditions. In the ostrich, Bonato et al. (2010) reported that re‐suspending ostrich semen to seminal plasma of the same male or different male was beneficial for maintaining sperm viability as compared to re‐suspending semen to Dulbecco's Modified Eagles Medium (a commercially manufactured cell culture medium). This further supports the insight that seminal plasma components such as proteins play a protective role in sperm cells. Therefore, further proteomics research is needed to characterise ostrich seminal plasma proteins and determine their roles in ostrich reproductive functionality (Including the production of fertile eggs after artificial insemination, which has previously been reported as a limitation; See Muvhali 2022) and sperm preservation protocols. On the other hand, since diet was confounded with season in this study, it could be that its effect on seminal plasma protein concentration was masked by season. Diet variation in metabolizable energy and protein composition has been reported to affect testicular size, testicular functionality, sperm quality, and subsequently seminal plasma composition in chickens and sheep (Ghorbankhani et al. 2015; Jiwuba et al. 2020). In ostriches, the effect of diet on semen characteristics and seminal plasma composition remains unclear but based on reports from other livestock species it could be expected that diet could have a similar effect and should be investigated in future. The frequency of semen collection had no effect on seminal plasma protein concentration, total seminal plasma proteins and seminal plasma volume. Seminal plasma volume was not influenced by season of semen collection in this study. This implies that semen can be collected relatively frequently without compromising seminal plasma protein composition, as well as in any season without a distinct alteration in seminal plasma volume. However, it is noteworthy to state that seminal plasma volume in the current study falls within the range for the ostrich recorded previously by Smith et al. (2018a).

Male ostriches in the present study differed significantly in most semen characteristics, as well as seminal plasma volume, seminal plasma protein concentration, and total seminal plasma proteins. In addition, repeatability estimates ranging from low to high were recorded for semen characteristics, while seminal plasma characteristics were moderately repeatable. Between‐male variation in ostriches for semen characteristics in this study is a well‐established phenomenon and has been widely reported in the literature (Bonato et al. 2010, 2011, 2014; Muvhali et al. 2020), suggesting that identification of males with favourable semen characteristics could be exercised for improving sperm quality. In addition, the repeatability estimates for semen volume and sperm concentration in this study appeared to be consistent with that reported previously by Cloete et al. (2015) for the same traits. Most notably, this study is the first to report repeatability estimates for ostrich seminal plasma protein concentration, seminal plasma volume, and total seminal plasma proteins. Although these results add to our previous estimates (Cloete et al. 2015; Muvhali et al. 2020, 2022b), the role it might play in current‐flock selection still needs verification. Since repeatability is seen as the upper limit of heritability, it also indicates that these traits may be heritable and amenable to selection. So far, the dedicated data needed to demonstrate the genetic basis of ostrich semen traits are lacking and dependent on future studies.

Commercial ostrich farming is characterised by different breeds and crosses within the production system. While the current study focused exclusively on the South African Black ostrich (common breed at the research facility), other breeds such as the Kenyan Red, Zimbabwean Blue, and crosses among these three breeds remain under‐researched in terms of both semen characteristics and seminal plasma protein composition. In chickens, breed effects on seminal plasma protein concentration have been documented (Santiago‐Moreno et al. 2019), highlighting the need for similar investigations across ostrich breeds and crosses in the future. Therefore, future studies should explore breed‐related differences in seminal plasma protein concentration and semen characteristics in other ostrich breeds.

## 5 Limitations of the Study

It is important to note that the literature cited on the ostrich, specifically the studies by Bonato et al. (Bonato et al. 2010, 2011, 2014), Smith et al. (2016), and Muvhali et al. (2020), were performed on the same ostrich population providing birds for this study. Therefore, results may vary in different ostrich populations and under alternative management conditions. Currently, there is limited availability of ostrich populations capable of providing routine physiologically relevant semen samples for extensive analyses. We would like to contend that the traits evaluated here provide a benchmark for future investigations using a larger sample size and utilising more diverse ostrich populations.

Further, we concede that the study is compromised by the confoundment of diet and season. However, drawing from our previous studies (Bonato et al. 2014; Muvhali et al. 2020), we would like to propose that our attribution of observed differences to mostly seasonal variation should be allowed to stand until it is confirmed or refuted by further, more comprehensive studies. We are also acutely aware of the fact that the contentions put forward are based on data from a limited number of males. Based on our previous studies on between‐male variation (Bonato et al. 2010, 2011, 2014; Smith et al. 2016; Muvhali et al. 2020), we feel that such inferences are quite robust across studies, adding credibility to the present results.

## 6 Conclusions

Season significantly influenced both the concentration and total seminal plasma proteins in the ostrich, with higher protein levels observed during winter, the period of lower sperm concentration and total sperm output. This pattern may indicate that seminal plasma proteins may play a compensatory role to mitigate reduced sperm quality. Future proteomics research is needed to characterise proteins present in the seminal plasma and to further investigate their functional activities in the ostrich. The frequency of semen collection did not have an influence on seminal plasma protein concentration and total seminal plasma proteins, indicating that seminal plasma protein reserves were not depleted by the collection intensity applied in this study. Lastly, between‐male variation and significant repeatability estimates in this study highlight the potential for genetic selection to improve seminal plasma protein concentration and total seminal plasma proteins in the ostrich.

## Author Contributions

**Pfunzo Muvhali:** formal data analysis and interpretation, writing – original draft, review and editing. **Maud Bonato:** conceptualization, project administration, design, writing – review and editing. **Irek Malecki:** conceptualization, design, writing – review and editing. **Kevin Douglas:** data collection. **Pieter Swart:** writing – review and editing. **Schalk Cloete:** conceptualization, design, funding acquisition, project administration, writing – review and editing.

## Funding

This work was supported by Western Cape Agricultural Research Trust.

## Conflicts of Interest

The authors declare no conflicts of interest.

## Data Availability

The data that support the findings of this study are available from the corresponding author upon reasonable request.
